# Diagnostic Aids in Pediatric Dentistry

**DOI:** 10.5005/jp-journals-10005-1073

**Published:** 2011-04-15

**Authors:** Gopakumar R, Manju Gopakumar

**Affiliations:** 1 Dean, Professor and Head, Department of Oral Medicine and Radiology, Mahatma Gandhi Dental College and Hospital Sitapura, Jaipur, Rajasthan, India; 2 Reader, Department of Pedodontics, AB Shetty Memorial Institute of Dental Sciences Mangalore, Karnataka, India

**Keywords:** Diagnostic aids, Disease, Treatment plan, Children.

## Abstract

Diagnosis involves development of a comprehensive and concise database of pertinent information, sufficient to understand the patient’s problem as well as answer questions arising in the treating clinicians’ mind. It is an accomplished art to develop a communication with the child and elicit relevant information from him. Thus, the signs and symptoms elicited on the basis of patient’s experiences and clinician’s knowledge forms the elementary framework of a good prognosis. This article aims to unveil the “must know” fundamentals of a sound diagnosis for a sound treatment plan.

## INTRODUCTION

Childhood is the period of life’s greatest physical, psychological and emotional growth; the child we see today is no longer the same tomorrow. The child patient presents a challenge to the dentist, who must solve the problems of today with an eye to the future and dental health of an adult.

Diagnosis is a process by which the practitioner distinguishes one disease from another, differentiates between normal and abnormal, and determines the etiology of abnormal conditions (Forrester). Accurate diagnosis can only be achieved by systematic and methodical collection of data. The present article illustrates the various diagnostic aids that can be used clinically in pediatric dentistry for detection and evaluation of commonly seen dental diseases.

The various diagnostic aids can be categorized as:

- Routine diagnostic aids
- Specialized diagnostic aids
- Advanced diagnostic aids.

*Routine diagnostic aids:* The clinical intraoral examination is performed systematically in a clean, dry, well-illuminated mouth using the mouth mirror, explorer and periodontal probe.

*Specialized diagnostic aids:* These are used for the diagnosis of specific dental problems like detection of dental caries, pulpal diseases and orthodontic problems.

## DIAGNOSIS OF DENTAL CARIES

Dental caries is a chronic disease that involves destruction of tooth structure, which can lead to loss of masticatory function and unesthetic appearance of affected enamel.^1^ The boundaries of caries diagnosis and caries interventions are changing.^1^ Dentists currently use visual, tactile and radiographic information to detect relatively advanced changes in the dental hard tissues. Diagnosis of dental caries is often regarded as synonymous with the detection of clinical signs of tissue damage caused by the disease, i.e carious lesions and cavities.^2^

### Methods of Clinical Diagnosis of Dental Caries

**Fig. 1:**
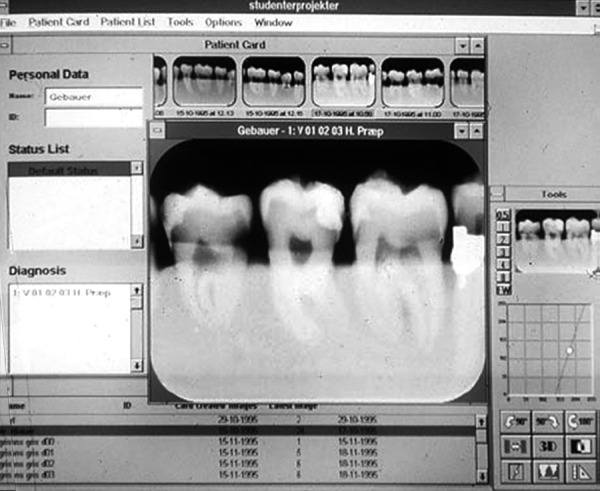
Digital radiography

Dental caries is a dynamic process and accurate diagnosis of the very incipient stages of a carious lesion can result in its reversal by the use of proper intervention methods:

- *Clinical method (visual-tactile method):* GV Black in1924 suggested the use of a sharp explorer to examine dental caries and the tooth surface was counted as decayed if slight pull was required to remove the explorer from the tooth surface. The same suggestion was given by Simon in 1956, Gillmore in 1982, and Marzouk and Sturdevant in 1985. Today it has been proved that the explorer point may fracture the demineralized enamel leading to cavitations. Use of a mirror and blunt probe is the most common method of diagnosing tooth decay. A sharp probe can break the intact tooth surface and one of the enamel lesions causing a cavity.^3^
- *Radiographic methods:* Radiographs can be classified into the conventional and advanced techniques. Though, conventional radiographs like bitewing and intraoral periapical radiograph are most frequently used for the detection of caries, they may cause overlapping of teeth due to faulty angulations and may also miss the initial lesion. During the primary dentition, the occlusal surface is most susceptible to caries attack, but with the eruption of first permanent molars the incidence of proximal lesions greatly increases. In such situation, bitewing radiographs are absolutely required to detect proximal lesions in primary molars.
  The Advanced radiographic techniques include digital radiography and xeroradiography. Digital radiography (Fig. 1) is a digital, filmless technique for intraoral radiography, utilizes very little of the radiation to which the patient has been exposed and avoids the need for developing films.^4^ Xeroradiography has the advantages of producing less radiation and edge enhancement along with its wide latitude of exposure.
- *Tooth separation:* In this method orthodontic modules or bands can be used to achieve slow separation and by separating the teeth one can visualize the proximal and approximal surfaces.

Recent Advances for Caries Diagnosis^5^

**Fig. 2:**
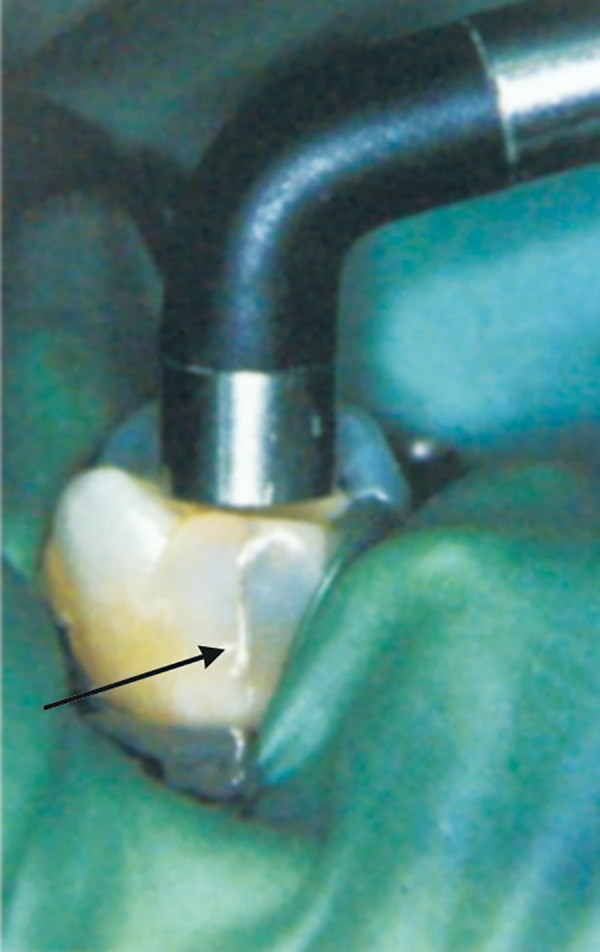
Staining and transillumination

*Fiber-optic transillumination* diagnoses approximal lesions in anterior teeth and posterior teeth by utilizing fiber-optic light source with the beam reduced to 0.5 mm in diameter (Fig. 2).

*Electrical resistance measurement* is a method of caries detection based on differences in the electrical conductance of carious and sound enamel. A comparative study was done regarding the accuracy of electronic caries monitor and visual diagnosis for the detection of occlusal dentine caries in primary teeth, and it was found that ECM did not provide increased accuracy over visual diagnosis when detecting occlusal caries in primary teeth.^6^

**Fig. 3:**
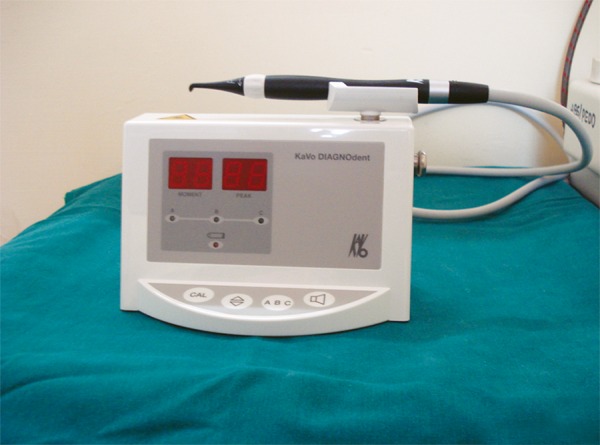
Optical method of fluorescence

*Laser fluorescence device* utilizes fluorescence and light scattering, where the visible light in the blue green region has been used as the light source for the detection of smooth surface and fissure caries at an early stage. A portable diode laser-based system was developed (Diagnodent), which is best suited for caries detection on occlusal and accessible smooth surfaces.^7^ A study done for detection of early carious lesions in primary molar teeth using Diagnodent, it was found that this method does not perform well in detecting initial enamel caries lesions.^8^ An *in vitro* study was done to determine the clinical efficiency of Diagnodent in detecting occlusal caries and it was found that Diagnodent (Fig. 3) is superior to visual and radiographic methods in diagnosing occlusal caries.^9,10^

*Caries detector dyes,* such as silver nitrate, methyl red and alizarin stain have been used to detect carious sites by change of color.

*Ultrasonics* utilizes a sonar device in which a beam of ultrasound waves is directed against the tooth surface and, if reflected, is picked up by an appropriate receiver. This method can be readily adopted to easily accessible areas but not for interpriximal surface.

Newer methods for caries detection include electroconductivity measurements (ECM), direct digital radiography (DDR), digital imaging fiberoptic transillumination (DIFOTI) and endoscopic filtered fluorescence method (EFF). According to Ten Cate et al in 1996, EFF is shown to be highly sensitive for occlusal caries in enamel but sensitivity is poor for occlusal caries in dentin.^11^

*Magnetic resonance microimaging* is a noninvasive technique wherein three-dimensional visualization of the carious lesion is possible and the extent of the carious lesions and its relation to other tooth structures can be assessed.

## PULPAL DIAGNOSTIC AIDS

Throughout the life of the tooth, vital pulp tissue continues to produce secondary or reparative dentin in response to biologic and pathologic stimuli. Pulp tissue keeps dentin supple and moist and, in turn, assures protection from forces of mastication.^12^

The diagnosis of dental pulp status should be seen as a synthesis of history, clinical examination, special tests, such as vitality tests, radiological examination and not as the outcome of one specific test.^13^

Most of the diagnostic tests, such as vitality tests used in conventional endodontic therapy are of very little, limited or no value in primary teeth and permanent immature teeth.^12^

The pediatric patients generally have a very low pain threshold compared to adults, so they cannot always describe subjective symptoms or sensitivity to a stimulus. As pulp vitality tests require the response of pain to stimulation, the results expressed can be exaggerated in a young patient due to failure of immature teeth to respond to the same. Since majority of children perceive the vitality testing methods as unpleasant stimuli, chances of false-positive or false-negative results are common in children. In primary teeth, due to the lack of development of the plexus of Raschkow in the pulp-dentin complex, pulp tests like thermal and electric tests are unreliable.^14^ So, they are used as an adjuvant to the other clinical diagnostic aids in dentistry.

To derive a correct diagnosis, a thorough recording of the chief complaint, medical history, dental history, extra-oral examination, intraoral examination and diagnostic tests are to be recorded and performed.

There are five basic directions toward which the clinician’s questioning is to be focused if the child reports of dental pain, which includes localization, commencement, intensity, provocation and duration.

*Extraoral examination* involves the observation of patient at the operatory, visual and palpation of the face, lymph nodes, etc. Intraoral examination includes both soft and hard tissue examination. Soft tissue examination includes observation of the gingiva, mucosa and tongue for any lesions, swellings and ulcers. Hard tissue examination should include visualization, palpation, mobility and percussion of teeth for any pathology and the use of diagnostic aids should follow, if necessary. These include mobility testing, thermal test, electric pulp test, staining and transillumination, anesthetic test, test cavity, bite test and conventional radiography.

All the available methods for assessment of pulpal vitality like electric pulp testing, application of thermal stimuli and the preparation of test cavities are indirect, and they rely upon the subject’s perception of peripheral nerve stimulation. Clinically, it is well-known that these tests suffer from varying degrees of unreliability.^13^

### Conventional Tests

*Mechanical tests* for pulp vitality include probing or blowing air, test cavity test, percussion tests, anesthetic test and occlusal pressure test.

**Fig. 4:**
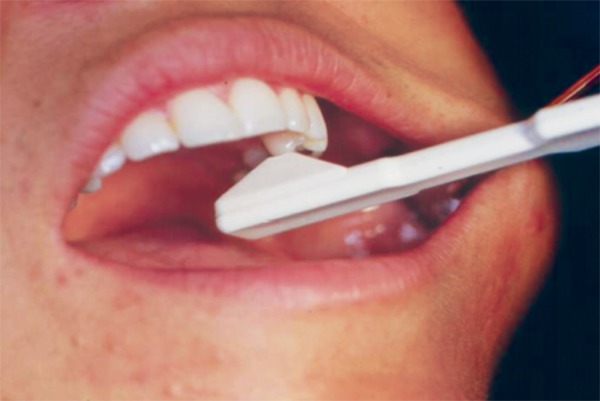
Tooth slooth for bite test

1. *Probing or blowing air:* It is a very simple method which appears to cause pain by initiating hydrodynamic pressure changes in the dentinal tubules, thus affecting the pulp.
2. *Test cavity:* Make a preparation through the enamel or the existing restoration until dentin is reached at a slow speed without a water coolant. If the pulp is vital, the heat from the bur will probably generate a response from the patient, however, it may not necessarily be an accurate indication of the degree of pulpal inflammation. Once a vital response is elicited, no further heat producing work should be performed.^15^ Test cavities are not reliable in nervous patients.
3. *Percussion:* A dull sound on percussion signifies abscess formation; a sharp sound signifies merely inflammation.^16^ Percussion test cannot be used in pediatric patients because it is difficult to get periodontal response due to resorption of deciduous roots, and there will be furcal involvement in long standing inflammation of deciduous tooth unlike in the apical area as in permanent teeth.
4. *Anesthetic test:* The anesthetic test can help to identify the quadrant from where the focus of pain originates. The suspected tooth should be anesthetized and, if the diagnosis is correct, the referred pain should disappear.
5. *Occlusal pressure test:* This test is useful in identifying teeth with symptoms of apical periodontitis, abscess or cracks. In patients with tooth infractions (cracked tooth syndrome) is pain often experienced when biting force is released rather than during the downward chewing motion (Fig. 4).^15^

**Fig. 5:**
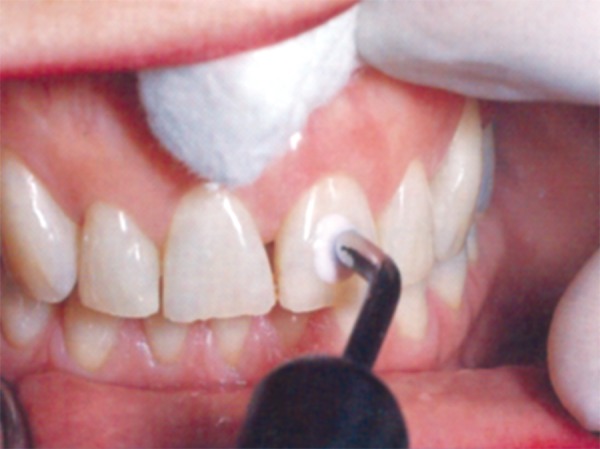
electrode of electric pulp tester

*Thermal Testing* is useful to assess the vitality of the pulp and to identify the offending tooth correctly in situations where the patient is unable to locate the source of the pain.^15^ A heat test is not a test for pulp vitality. An abnormal response to heat usually indicates the presence of a pulpal or periapical disorder that requires endodontic treatment.^17^ There are some limitations for the use of heat test in pediatric patients as it might increase the anxiety of the child. Secondly, the heat might damage the pulp, since pulp horns are highly placed in deciduous teeth and if the child is uncooperative the heat might cause injury to the soft tissue.

In cold test, the application of cold can be done by many methods like a stream of cold air, ethyl chloride, application of ice, dichlorodifluoromethane and CO_2_ snow. A response to cold indicates a vital pulp, regardless of whether the pulp is normal or abnormal.^17^ In pediatric patients, application of CO_2_ snow produces a low intrapulpal pressure and is far more effective and reliable even in immature tooth.

**Fig. 6:**
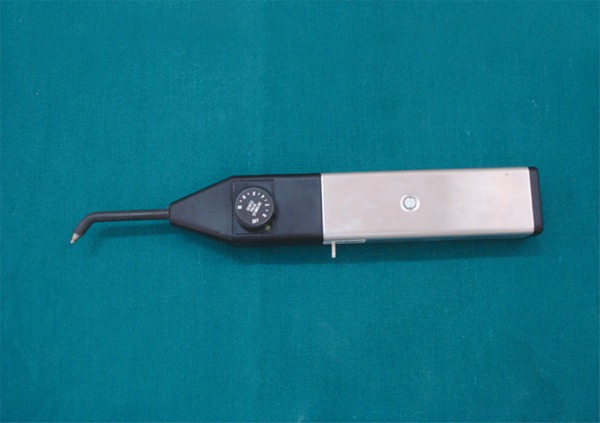
Electric pulp tester

Electric pulp test utilizes the tip of a testing probe (Fig. 5) that is coated with water or petroleum based media. The coated tip is placed in the incisal third of the facial or buccal area of the suspected tooth (Fig. 6) to be tested and the pain response is elicited. A false-positive response is obtained in cases when the electrode contacts the gingiva, liquefaction necrosis, failure to isolate and dry the teeth properly and multirooted teeth where the pulp may be vital in one or more root canals. A false-negative response is seen in patients with heavily premedicated, a recently traumatized tooth, teeth with an immature apex, excessive calcification in canals and the presence of pulp protecting bases.

Electric pulp testing has shown to be unreliable or rather non effective in deciduous teeth and immature permanent teeth because the relationship between odontoblasts and nerve fibers of the pulp has yet to develop.^18^ Failure of immature teeth to respond to the electric pulp testing may be caused by the lack of development of Raschkow plexus in the region of pulp dentin border. Another reason for the unreliability of electric pulp test in deciduous teeth is that the nerve fibers are the last to develop and first to degenerate in these teeth.

The advanced pulpal diagnostic aids include laser Doppler flowmetry, pulse oximetry, dualwave spectro-photometry, plethysmography, liquid crystal testing, time-temperature graph, electronic thermography, ultrasonic imaging, xeroradiography, digital imaging, subtraction radiography and computed tomography.

The laser Doppler flowmetry technique is a noninvasive, electro-optical technique, which has been shown to have a potential of assessing the vitality of teeth by detecting the presence or absence of pulpal blood flow. Studies were carried out to compare LDF with conventional pulp tests, EPT (electric pulp testing) and thermal tests, in children with certain dental injuries. It was concluded that LDF identified more vital and nonvital teeth correctly at earlier time periods following injury than conventional tests.^19^ Evans et al found LDF to be a reliable method for assessing the pulpal status of traumatized anterior teeth than standard pulpal diagnostic tests.^20^

*Pulse oximetry^8^* is a noninvasive technique which determines the percentage of O_2_ saturation of circulating arterial blood by the placement of the probe in the middle third of the crown. Goho C^8^ (1999) evaluated the efficacy of pulse oximetry for testing pulp vitality for primary teeth and immature permanent teeth, and concluded that pulse oximetry is an objective, atraumatic clinical alternative to the present electrical and thermal methods of assessing pulp vitality in children’s teeth.

*Dual wavelength spectrophotometry^8^* is a method independent of pulsatile circulation, which detects the presence or absence of oxygenated blood at 760 and 850 nm. This method is noninvasive, inexpensive and do not rely on subjective patient response, and therefore yields objective results. The limitation is that it detects only the presence of hemoglobin not the circulation of blood.

*Plethysmography^8^* is a potential noninvasive method to detect vascularity within the dental pulp. Its advantages include less signal contamination derived from periodontal blood flow and less signal noise (PDL blood flow) compared to LDF due to the pathway of the light (transmitted light).

*The time-temperature graph method^8^* is a concept of diagnosing tooth vitality by temperature measurement and can provide valuable information on the integrity of the underlying pulp.

*Electronic thermography^8^* produces color images of the body that indicate relative differences in temperature in both superficial and deep areas. A study by Pogrel and Yen was done to assess the vitality of 20 teeth including necrotic pulps, root canal fillings and normal pulps by using infrared thermography, and showed that when the teeth were cooled by air spray to approximately 22°C and then allowed to re-warm to their original resting temperature of 29°C, the teeth containing normal pulp took about 5 seconds, whereas necrotic and root canal filled tooth took about 15 seconds.^9^

*Optical reflection vitalometer* is a system based on pulse oximetry, but the difference from conventional pulse oximetry is that adsorption is measured from reflected light instead of transmitted light. Preliminary tests showed that vital and nonvital pulposus reflected the radiation differently.

*Ultravioletfluorescence* is a test of vitality which accentuates the color changes occurring in a tooth, when pulp is damaged by trauma or inflammation.

## ORTHODONTIC DIAGNOSTIC AIDS

The responsibility of early detection and management of developing malocclusion rests with the pedodontists because they see the patient at a very young age at various intervals like preschool age, school age and the teenage period. Diagnosis requires the collection of an adequate database of information about the patient and distillation from that database of a comprehensive but clearly stated list of the patient’s problems. The database may be thought of as derived from three major sources:

- Patient questioning
- Clinical examination of the patient
- Evaluation of diagnostic records.

### Questionnaire/Interview

Questionnaire/Interview records the initial patient contact details, chief complaint, medical history, dental history, genetic history, social-behavioral history, age, sex, prenatal history and family history.

### Clinical Examination of the Patient

This includes an extraoral examination which will record the general health, body type (ectomorphic, mesomorphic, endomorphic), posture, and the physical growth status. Facial features include that facial type (mesofacial, brachyfacial and dolichofacial), shape of the head (dolichocephalic, mesocephalic, brachycephalic), profile analysis (anteroposterior and vertical relationship), lip posture at rest (color, size, mentolabial sulcus) and relative symmetry of facial structures (size and shape of nose, chin button size and contour).

Intraoral examination should record the jaw relationship (anterior-posterior relationship, vertical relationship, lateral relationship), open mouth examination of teeth, soft tissue appraisal and functional assessment (respiration, speech difficulties indicating dental problems, differential diagnosis of swallowing types, occlusal interference).

### Evaluation of Diagnostic Records

Three Major Categories:

1. *Evaluation of teeth and oral structures:* The general guideline is that any medical problems, dental caries or pulpal pathology and periodontal disease must be under control before orthodontic treatment begins.
2. *Occlusal evaluation:* Three aspects require evaluation-mastication and swallowing, speech and TMJ problems.
3. *Evaluation of facialproportions:* Three step examination:
  - Macro esthetics―‘Face in 3D space’. For example, asymmetry, excessive/deficient facial height, mandibular excess/deficiency.
  - Mini esthetics―‘Smile framework’. Evaluation of excessive gingival display on smiling, inadequate anterior tooth display, excessive buccal corridors, etc.
  - Micro esthetics―‘The teeth’. Assessment of tooth proportions in height and width, gingival shape and contour, connectors and embrasures, black triangular holes and tooth shade.

## ANALYSIS OF DIAGNOSTIC RECORDS

1. Cast analysis: Mixed dentition model analysis
2. Cephalometric analysis
3. Diagnostic radiographs and photographs
4. Orthodontic classification.

### Mixed Dentition Model Analysis

Model analysis is a valuable tool in orthodontic diagnosis and treatment planning as it provides a 3D view of arches and helps in early assessment of available space. Mixed dentition model analysis evaluates the amount of space available in the arch for succeeding permanent teeth and the necessary occlusal adjustments. Nonradiographic method include Moyer’s analysis, IOWA’s prediction method, Johnson and Tanaka’s analysis, Boston university approach, regression equation, Ballard, Wyllie and Owen’s analysis. Radiographic methods include Nance analysis, Hixon-Oldfather analysis, Staley-Kerber’s analysis, Huckkaba analysis, etc.

### Cephalometric Analysis

**Fig. 7:**
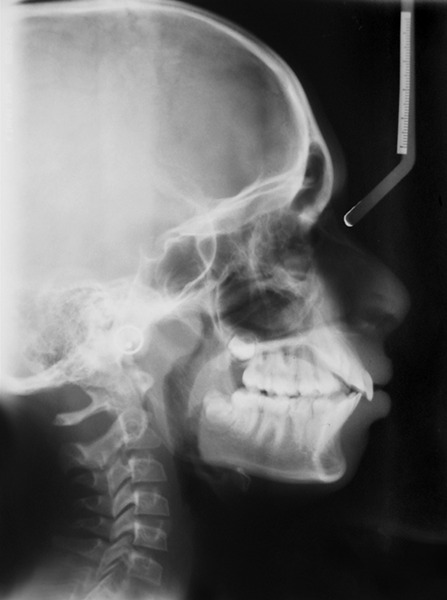
Cephalogram

Radiographic cephalometry (Fig. 7) is the measurement of head from bony and soft tissue landmarks on the radiographic image.^12^ Lateral cephalograms reveal the details of skeletal and dental relationships that cannot be observed in other ways, and they allow a precise evaluation of the response to treatment.

**Fig. 8:**
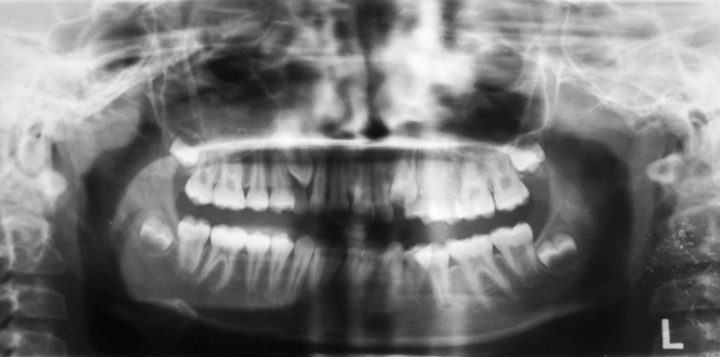
Orthopantomograph for orthodontic diagnostic purposes

### Diagnostic Radiographs and Photographs

Clinical photographs and diagnostic radiographs help in assessing the visual treatment objective. Figure 8 shows an orthopantomograph for orthodontic diagnostic purposes.

### Orthodontic Classification

Angle’s 1899 classification was based on identifying occlusal relationships and classifying a malocclusion according to the position of the mandibular first molar and its relationship to the opposing maxillary first molar. It classifies malocclusion into Angle’s class I, class II (div 1, div 2, subdivison) and class III relations (pseudo class III, subdivision).

## MATURITY INDICATORS

1. *Neural age* helps us to understand that the patient is mentally developed to understand the need for the treatment, to what extend he can cooperate and follow instructions.
2. *Mental age* is an index of maturation of the mind, and increases at a rate that depends on many intrinsic and environmental factors. Some of the performance tests used to measure intelligence are the Standard-Binnet test and Wechsler scale.
3. *Physiological and Biochemical age* are a series of physiological and biochemical changes occurring during growth, which can be correlated to skeletal and chronological age.
4. *Chronological age* is determined by passage of time since birth, which is a poor indicator of maturity.
5. *Sexual/Pubertal age* is the stage of development of secondary sexual characteristics, and provides a physiological calendar of adolescence that correlates with the individual’s physical growth status.
6. *Dental age* is determined by formation or eruption of teeth.
7. *Skeletal/Radiological/Anatomic age* is considered to be the most reliable age for growth assessment for orthodontic purposes. It is closely related to the growth of the individual.

### Anatomical Regions

**Fig. 9:**
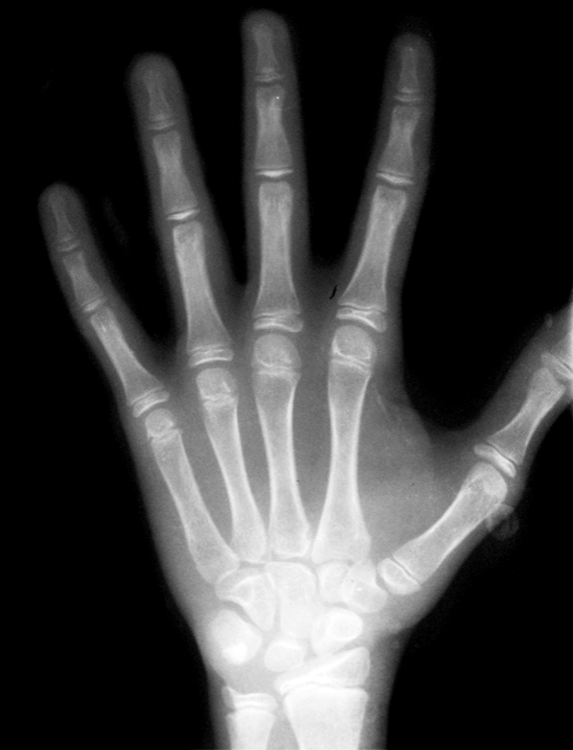
Anatomy of hand wrist radiographs

Regions normally used for assessing growth and development include: Head and neck (skull, cervical vertebrae), upper limb (shoulder joint-scapula, elbow, hand wrist and fingers― Fig. 9) and lower limb (femur and humerus, hip joint, knee, ankle, foot―tarsals, metatarsals).

## COMPUTERIZED DIAGNOSTIC SYSTEMS

Digital cephalometric involves digitization, which is a form by which analog information is converted to digital form and are recorded and stored in a data set. This data set is starting point for the formulation of various computer generated visual treatment objectives. Video cephalometry which includes digitization of the cephalogram followed by sizing the profile video image to the cephalogram. Digital photography enables storage of images in a digital form on a storage media and is a simple aid to transfer and manipulate such data. Three-dimensional imaging techniques provide extensive possibilities for the detailed and precise analysis of the whole craniofacial complex, for virtual (on-screen) simulation and real simulation of orthognathic surgery cases on biomodels before treatment as well as for the detailed evaluation of the effects of treatment. Cone beam volumetric tomography uses a cone-shaped X-ray beam with a special image intensifier and a solid state sensor or an amorphous silicon plate for capturing the image. Other supplemental diagnostic aids include occlusograms which are tracings of a photograph or a photocopy of a dental arch, and can be used to estimate occlusal relationships along with arch length and width. Electromyography is a procedure used for recording the electrical activity of muscles. It detects abnormal muscle activity associated with certain forms of malocclusion.
